# Supplementary material for: Prevalence and associated factors of premenstrual syndrome among women of the reproductive age group in Ethiopia: Systematic review and meta-analysis
Source: PLoS One. 2020 Nov 6;15(11):e0241702. doi: 10.1371/journal.pone.0241702 (PMC7647055; doi:10.1371/journal.pone.0241702)
Supplement: Attachment — Submitted filename: Response to Reviewers.docx [file pone.0241702.s004.docx]

**Rebuttal letter**

**Manuscript ID number:** PONE-D-20-03722

**Prevalence and associated factors of premenstrual syndrome among women of reproductive age group in Ethiopia: systematic review and meta-analysis**

We would like to express our heartfelt gratitude to editor and reviewers for constructive comments and guidance which are extremely helpful to improve this manuscript. Here are point-by-point responses for the comment raised. We had thoroughly revised the manuscript and provided the amendments that have been made to the manuscript text.

**Response to Editor comments**

1. The manuscript thoroughly edited based on PLOS ONE's manuscript writing format.
2. The data from which result and conclusion drawn was attached as one of supporting information.
3. The captions for Supporting Information files were written at the end of manuscript

**Response to Reviewer 1 Comments**

1. Similarity of Figure 1 on flow chart showing articles selection strategy with previous published work was resolved.
2. English language edition was done by language editor.

**John Odjenimah**, **email: ighoyiwi25@gmail.com**

**Response to Reviewer 2 Comments**

1. A. The definition of premenstrual syndrome was clarified.

B – E. Comment on this sub – section concerning diagnosis of PMS is very important but the scope of the current study not extends up to the level of evaluating diagnostic tools. As describe in the manuscript, all primary studies included in the current study were cross sectional studies. Those studies done by using two standard tools to diagnosis PMS such as American College of Obstetrics and Gynecology (ACOG) and Diagnostic and Statistical Manual of mental disorders IV (DSM-IV).

For clarification on diagnosis of PMS by using ACOG; diagnostic criteria were described in paragraph two in introduction section.

2. All the indicated (subsection A -K ) and other section of manuscript were edited for English language by language editor.
3. Independent variables

I. Age at menarche was divided into two groups: those 12 or younger and those older. This is classification done by primary studies. Indeed, it has limitation to describe association with PMS. Thus, we described this as limitation of study on manuscript.

II. Dividing menstrual pattern as regular or irregular was based on women’s report which is not based on record during each menses. All the study available on this title were cross sectional. So, we indicated recall bias as one of limitation of study.

4. A. The figures were edited accordingly and possible amendments were done. Figure 2 is forest plot that shows prevalence of PMS in Ethiopia. The labeled 84.7 is maximum confidence interval level for prevalence of PMS. Figure 3 also show prevalence of PMS in different settings. In both forest plots, dotted line is pooled prevalence and forests shows how weighted prevalence from each study spread around pooled prevalence.

Figure 4 shows odds ratio of associated factors. It may be mis-communication, there is no prevalence indicated as less than zero in manuscript.

B. Diagnostic criteria of PMS was described in paragraph two of introduction section.

C. This comment was applied in the different sections of manuscript

5. Language edition to whole part of manuscript was done
