# Supplementary material for: Prevalence and associated factors of premenstrual syndrome among women of the reproductive age group in Ethiopia: Systematic review and meta-analysis
Source: PLoS One. 2020 Nov 6;15(11):e0241702. doi: 10.1371/journal.pone.0241702 (PMC7647055; doi:10.1371/journal.pone.0241702)
Supplement: Attachment — Submitted filename: decision.docx [file pone.0241702.s005.docx]

**Dear Prof. Nülüfer Erbil,**

Greetings to you!

The authors of the manuscript titled ‘Epidemiology of Premenstrual Syndrome (PMS)-A Systematic Review and Meta-Analysis Study’ have satisfactorily carried out the revision to their manuscript.

The manuscript may be accepted for publication as the work carried out by Teshome Gensa Geta and the co-authors is of importance as it portrays women health across the globe.

Thank you.

Yours Sincerely

Padmanabhan Anbazhagan
