# Supplementary material for: Prevalence and associated factors of premenstrual syndrome among women of the reproductive age group in Ethiopia: Systematic review and meta-analysis
Source: PLoS One. 2020 Nov 6;15(11):e0241702. doi: 10.1371/journal.pone.0241702 (PMC7647055; doi:10.1371/journal.pone.0241702)
Supplement: Attachment — Submitted filename: response to reviewer.docx [file pone.0241702.s006.docx]

**Rebuttal letter**

**Manuscript ID number: PONE-D-20-03722**

Prevalence and associated factors of premenstrual syndrome among women of reproductive age group in Ethiopia: systematic review and meta-analysis

We would like to express our heartfelt gratitude to the editor and reviewers for constructive comments and guidance which are extremely helpful to improve this manuscript. We have a great appreciation for your in-depth insight. Here are point-by-point responses for the comment raised. We did a brief revision of the manuscript as per comments and provided the amendments that have been made to the manuscript text. The track change was done for the word changes and added sentences were red-colored on the revised manuscript with track changes.

**Response to Reviewer Comments**

1. Two words with type error have been corrected

Line 60: relived is replaced by relieved

Line 64: cycle is replaced by cycles’

2. Clarification on quality assessment and selection of articles has been done by adding the following sentence.

Line 127 in quality assessment under method’s sections: Those studies with high quality (quality score of 6 and more) were included in the analysis

Line 178 in study characteristics under result section: All the studies were published articles and their quality score range from 6 to 9 out of 10 points.

3. Line 295, comment on the word usage was considered and the appropriate term was replaced

Line 296: suffering is replaced by experiencing

Thank you for your diligence!

Teshome Gensa Geta, corresponding author
